# Design of Distributed Drive Control Strategy for Wheel Side Rear Drive Electric Bus Based on Neural Network Algorithm

**DOI:** 10.3389/fbioe.2022.905695

**Published:** 2022-05-31

**Authors:** Huipeng Chen, Yingjie Zheng, Sen Chen, Shaopeng Zhu, Jian Gao

**Affiliations:** ^1^ School of Mechanical Engineering, HangZhou DianZi University, Hangzhou, China; ^2^ College of Energy Engineering, Zhejiang University, Hangzhou, China

**Keywords:** electric bus, yaw moment control, neural network PID, distributed drive, lateral stability

## Abstract

The electric bus driven by the wheel-side motor has a natural advantage for the driving stability control of the vehicle because the torque of each driving wheel is independently controllable. Based on the advantages of the artificial neural network control algorithm, this paper designs a direct yaw torque control strategy based on neural network PID control, and combines the steering characteristics of the vehicle to distribute the driving torque. The co-simulation results of Matlab/Simulink and TruckSim show that the designed control strategy can effectively reduce the vehicle’s center of mass slip angle and lateral acceleration under medium and high speed conditions, and ensure the stable driving of the bus.

## Introduction

Distributed drive electric vehicles can achieve power and maneuverability that cannot be achieved by traditional driving methods, and have a wider management space in terms of economy and safety control, and have great potential (Gasbaoui and Nasri, 2012). But the relevant research and marketing is not easy. The difficulty in the design, implementation and optimization of the distributed drive electric vehicle control system is that the vehicle has multiple drive motors, which belong to the overdrive system and have redundant degrees of freedom. Each actuator affects each other and each force is coupled with each other, which belongs to the nonlinear system dynamics problem (Kiumars et al., 2012). The main purpose of the research on distributed drive electric vehicle is to improve the stability, safety and energy saving effect of the vehicle. From the perspective of torque distribution, one or more of motor energy efficiency, torque coordination and vehicle dynamics are considered to improve vehicle performance as a whole.

The stability-based distributed drive electric vehicle torque distribution strategy mainly considers the control of body yaw moment. This control strategy is relatively mature in traditional internal combustion engine-driven vehicles, and has been widely used in industrial applications. Following the idea of ​​chassis control, strategies such as ESP and AFS have been widely studied in distributed drive electric vehicles (Fujimoto et al., 2006; Xibo Yuan et al., 2012; Yuan et al., 2012; Wu et al., 2013), and the vehicle stability can be improved by controlling the distributed motor torque or the front wheel steering mechanism. This type of control system is mostly composed of a motion tracking layer and a torque distribution layer. The research content includes the acquisition of vehicle ideal state parameters, the design and implementation of motion tracking strategy and generalized force distribution strategy, etc. The related research is mostly based on this idea.

The acquisition of vehicle ideal state parameters is the basis of vehicle stability control. By introducing the ideal model of the vehicle driving process, the ideal vehicle stability state parameters can be derived, which is of great value to the handling and stability control of the vehicle (Mokhiamar and Abe, 2006; Yu et al., 2013; Yang et al., 2014). Considering the influence of the road adhesion coefficient, in order to avoid the vehicle running in a nonlinear state, it is necessary to constrain the limits of the yaw rate and the center of mass slip angle, thereby improving the stability control effect of the vehicle under extreme driving conditions (Fujimoto et al., 2004; Fujimoto et al., 2006; Xia, 2007; Jonasson et al., 2011).

At present, the algorithms for vehicle stability control mainly include fuzzy control, sliding mode control and PID control (Yi, 2020, Zou et al., 2009). However, the driving conditions of the vehicle are complex and changeable, and the mass and the position of the center of mass of the passenger car will change due to different driving conditions. The control algorithm mentioned above is less applicable to the operating conditions due to its own limitations. The artificial neural network control algorithm produced on the basis of modern neurology, biology, psychology and other disciplines can reflect the basic process of biological nervous system processing external things, and it is a calculation developed on the basis of simulating human brain nerve tissue. The system is a network system composed of a large number of processing units through extensive interconnection. It has the basic characteristics of the biological nervous system, and reflects some reflections of the human brain function to a certain extent. It is a kind of simulation of the biological system. The advantages of parallel, distributed processing, self-organization, and self-learning are widely used in many fields such as image recognition, computer vision, and control system design.

In this paper, the distributed rear-drive electric passenger car is taken as the research object, and the neural network PID control algorithm is designed based on the principle of direct yaw moment to improve the lateral stability of the vehicle. The main work includes the following three points: 1) A distributed drive control system with a hierarchical control structure is proposed for rear-drive electric buses, including a yaw moment formulation layer and a driving torque distribution layer. 2) In the yaw moment formulation layer, the neural network PID control algorithm is designed based on the direct yaw moment control principle. The control variable of the controller, the output is the optimal yaw moment under the current working condition. 3) Using Matlab/Simulik software to build the control strategy model and the bus model in TruckSim software, and build a joint simulation platform. The control strategy designed by the analysis is verified under different operating conditions.

## Build Dynamics Models

In order to design the adaptive distributed drive control strategy and verify it, it is necessary to establish a two-degree-of-freedom vehicle reference model, a five-degree-of-freedom vehicle dynamics model, and a TruckSim vehicle model.

### Reference Model

**FIGURE 1:**
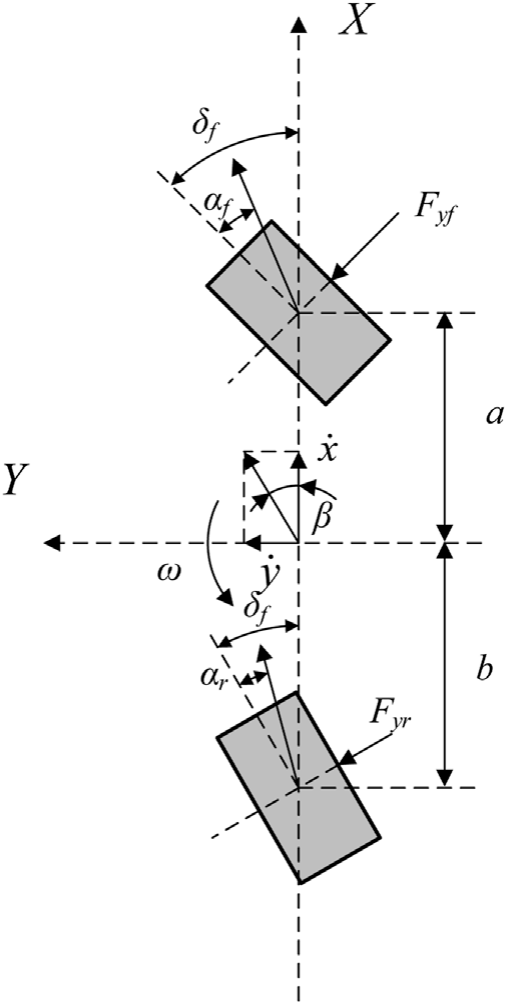
Two-degrees-of freedom vehicle model.

It is assumed that the longitudinal speed and lateral acceleration of the vehicle remain unchanged during the driving process, while the motion of the body in the pitch, roll and vertical directions are ignored, and only the two-degrees-of freedom of lateral motion and yaw motion are considered, As shown in Figure 1, *O* is the center of mass of the vehicle, *φ* is the yaw angle; *F*_*y*_ and *F*_*yr*_ are the lateral force received by the front wheel and the lateral force received by the rear wheel in the tire coordinate system, respectively; *δ*_*ij*_ is the tire rotation angle, and *I*_*z*_ is the rotation of the vehicle Inertia; *a* and *b* are the distances from the center of mass of the car to the front and rear axles respectively; $\dot{x}$ is the longitudinal vehicle speed; $\dot{y}$ is the lateral vehicle speed.

Taking the sideslip angle and yaw rate as state variables, the state equation of the system is as follows:

$$\left\{ \begin{array}{l} \dot{X}=AX+BU+CW\\ Y=X \end{array}\right.$$ (2.1)

And

$$\dot{X}=\left[\begin{array}{c} \dot{\beta }\\ \dot{\omega } \end{array}\right],X=\left[\begin{array}{c} \beta \\ \omega \end{array}\right],U=\text{Δ}M_{\omega },W=\left[\begin{array}{c} \delta_{f}\\ \delta_{r} \end{array}\right],A=\left[\begin{array}{cc} -\frac{C_{yf}+C_{yr}}{m\dot{x}} & \frac{bC_{yr}-aC_{yf}}{m{\dot{x}}^{2}}-1\\ \frac{bC_{yr}-aC_{yf}}{I_{z}} & -\frac{a^{2}C_{yf}+b^{2}C_{yr}}{I_{z}\dot{x}} \end{array}\right],B=\left[\begin{array}{c} 0\\ \frac{1}{I_{z}} \end{array}\right],C=\left[\begin{array}{cc} \frac{C_{yf}}{m\dot{x}} & \frac{C_{yr}}{m\dot{x}}\\ \frac{aC_{yf}}{I_{z}} & -\frac{bC_{yr}}{I_{z}} \end{array}\right]$$ (2.2)

In the above formula, *m* is the mass of the vehicle; *I*_*z*_ is the moment of inertia of the vehicle; *C*_*yf*_ and *C*_*yr*_ are the lateral stiffnesses of the front and rear wheels, respectively, which are positive values.

There are the following equivalents during the vehicle steady driving state.

$$\{\begin{array}{c} \dot{\omega }=0\\ \dot{\beta }=0 \end{array}$$ (2.3)

Combined Eq. 2.1 and Eq. 2.2, we can get:

$$\{\begin{array}{c} \omega_{d}=\frac{v_{x}}{L\left(1+Kv_{x}^{2}\right)}\delta_{f}\;\;\\ \beta_{d}=\frac{bLC_{r}+mav_{x}^{2}}{L^{2}C_{r}\left(1+Kv_{x}^{2}\right)}\delta_{f} \end{array}$$ (2.4)

In addition, considering the limitation of the road surface adhesion coefficient, when the steady-state values of the yaw rate and the vehicle sideslip angle derived from the linear two-degree-of-freedom reference model exceed the maximum value that the road surface can provide, the current road surface can provide as the expected value. On a road with a pavement adhesion coefficient of ***μ***, the boundary values of the yaw rate and the vehicle side slip angle (King and Mamdani, 1977; Rajamani, 2016; Chen et al., 2022a; Chen et al., 2022b) are calculated respectively.

$$\{\;\begin{array}{c} \omega_{bound}=0.85\frac{\mu g}{v_{x}}\\ \left|\beta_{bound}\right|\leq \tan^{-1}\left(0.02\mu g\right) \end{array}$$ (2.5)

Combined Eq. 2.3 and Eq. 2.4, we can get

$$\{\begin{array}{c} \omega_{d}=min\left\{\frac{v_{x}}{L\left(1+Kv_{x}^{2}\right)}\delta_{f},\omega_{bound}\right\}\\ \beta_{d}=min\left\{\frac{bLC_{r}+mav_{x}^{2}}{L^{2}C_{r}\left(1+Kv_{x}^{2}\right)}\delta_{f},\beta_{bound}\right\} \end{array}$$ (2.6)

In the above formula, *a* and *b* are the distance from the center of mass of the vehicle to the front and rear axles, *L* is the wheelbase, $C_{f}$ and $C_{r}$ are the cornering stiffness of the front and rear axles of the vehicle, $J_{Z}$ is the moment of inertia of the vehicle around the *Z* axis, $v_{x}$ is the longitudinal speed of the vehicle, ***μ*** is the pavement adhesion coefficient, and $\delta_{f}$ is the front wheel steering angle of the vehicle. In addition, *K* is the stability factor, which is an important parameter that characterizes the steady-state response of the vehicle. The value of *K* is obtained by the following Eq. 2.6.

$$K=\frac{m}{L^{2}}\left(\frac{a}{C_{r}}-\frac{b}{C_{f}}\right)$$ (2.7)

### Vehicle Model

In order to design and verify the distributed drive control strategy, it is necessary to establish a wheel-side rear-drive electric bus model.

**FIGURE 2:**
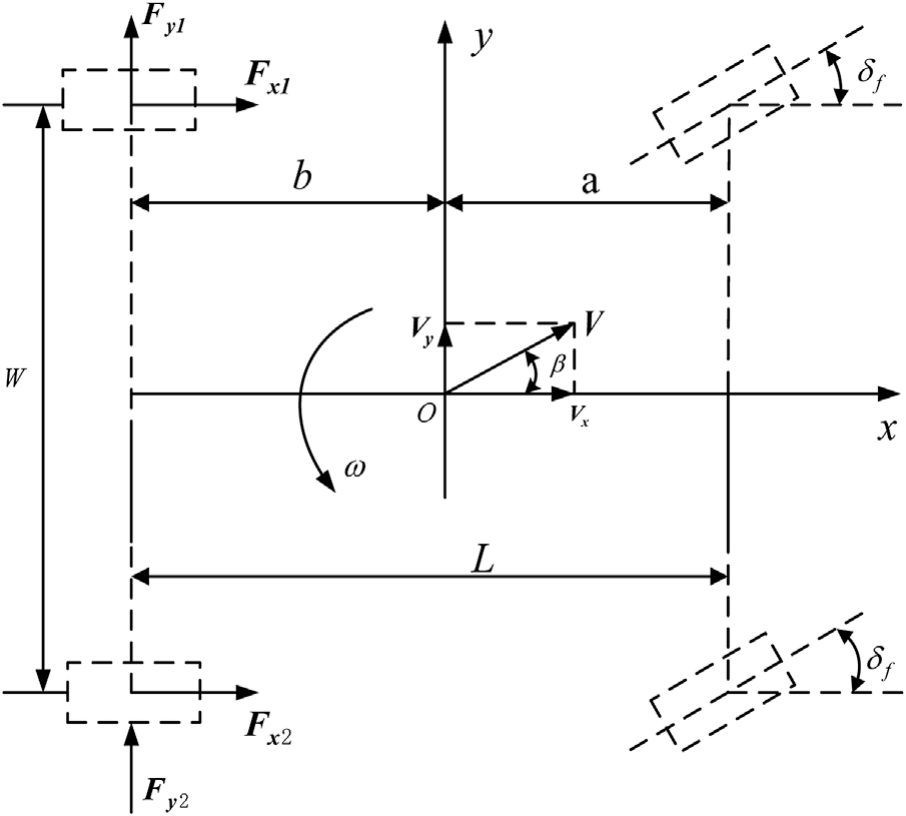
Five-degrees-of-freedom vehicle model.

This article mainly studies the lateral driving stability of electric bus, ignoring the pitch and roll motion of the vehicle, and only considers the five-degrees-of freedom of the vehicle’s lateral, longitudinal, yaw and the rotation of the four wheels. As shown in Figure 2, the simplified five-degree-of-freedom model can be used to analyze the rear-drive forces of the vehicle in direct yaw-moment control.

Longitudinal motion equation.

$$m\left({\dot{V}}_{x}-V_{y}\omega \right)=F_{x1}+F_{x2}$$ (2.8)

Lateral motion equation

$$m\left({\dot{V}}_{y}+V_{x}\omega \right)=F_{y1}+F_{y2}$$ (2.9)

Yaw motion equation

$$J_{Z}\omega =\frac{w}{2}\left(F_{x2}-F_{x1}\right)-b\left(F_{y1}+F_{y2}\right)$$ (2.10)

In the above formula, *m* is the mass of the vehicle; $V_{x}$ is the longitudinal velocity of the vehicle; $V_{y}$ is the lateral velocity of the vehicle; $J_{Z}$ is the moment of inertia of the vehicle around the *Z* axis; ***ω*** is the vehicle yaw rate; *a* is the distance from the center of mass to the front axle; *b* is the distance from the center of mass to the rear axle; ***w*** is the rear axle track; $F_{x1}、F_{x2}、F_{y1}$ and $F_{y2}$ are respectively the longitudinal and lateral reaction forces of the left and right driving wheels on the ground.

The wheel rotation dynamics equation can be obtained as follows.

$$I_{w}{\dot{w}}_{r}=T_{d}-T_{b}+F_{x}R$$ (2.11)

$I_{w}$ is the rotational inertia of the wheel; *R* is the wheel rolling radius; $F_{x}$ is the wheel friction; $T_{d}$ is the wheel driving torque; $T_{b}$ is the wheel braking torque.

TruckSim vehicle dynamics simulation software is used to build a complete electric bus model. TruckSim can build a nonlinear vehicle model in a parameterized manner as a control strategy simulation verification platform. Some parameters of the electric bus model are shown in Table 1.

**TABLE 1:** Reference values for some parameters of electric bus.

The name of the Parameter	The Reference Value	Unit
Vehicle mass (m)	12,800	kg
Length*Width*Height	12,000*2,500*3,150	mm
Height of the center mass (h)	1,200	mm
The center of mass to the front axle distance (a)	3,240	mm
The center of mass to the rear axle distance(b)	1,260	mm
Wheelbase (L)	4,500	mm
The front tire cornering stiffness $C_{f}$	119,283.4	N/rad
The rear tire cornering stiffness $\;C_{r}$	225,781.4	N/rad
Rear wheel pitch (W)	1863	mm

## Distributed Drive Control System Design

**FIGURE 3:**
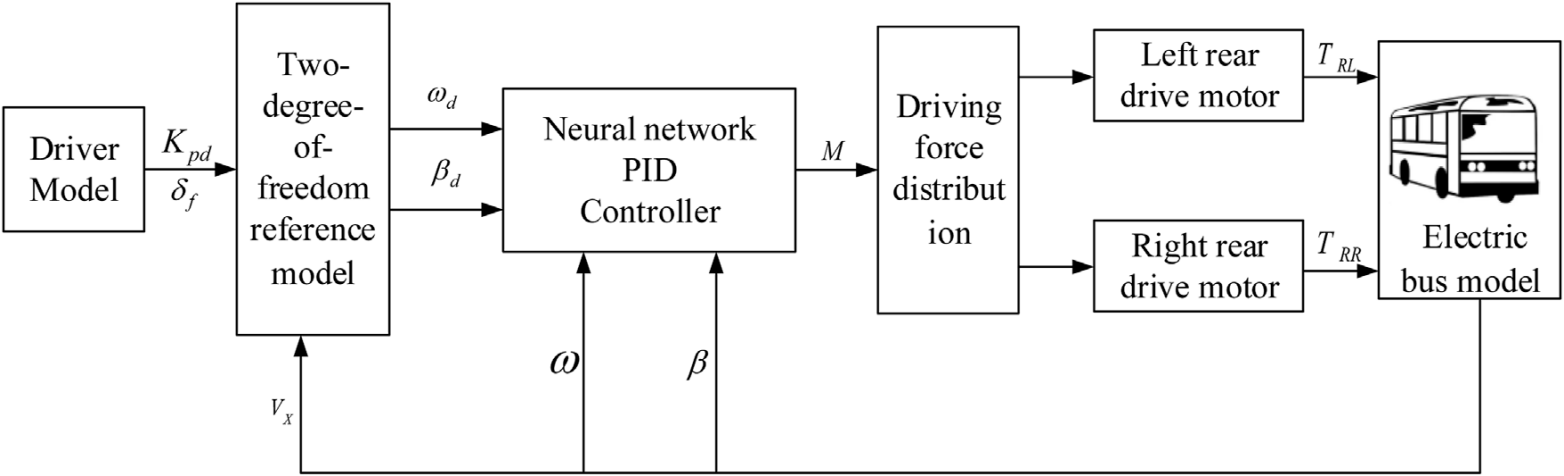
Direct yaw moment control flow chart.

For the distributed rear-drive electric bus, by designing a reasonable driving force control strategy according to various driving conditions can improve the driving stability and driving safety of the bus. Based on the principle of yaw moment, a rear wheel drive force distribution control strategy to maintain the body attitude and driving route of electric bus is designed. The control strategy structure is divided into two layers: yaw torque setting layer and driving torque distribution layer, as shown in Figure 3.

The desired yaw moment setting layer inputs the received driver information $\delta_{f}$ and $K_{pd}$ into the reference vehicle model to obtain the expected yaw rate and sideslip angle. The expected yaw rate and sideslip angle to the actual yaw rate and side slip angle are compared, and the desired yaw moment is obtained by the Neural network PID controller and input to the next layer. In the driving force distribution layer, the driving forces are reasonably distributed to the left and right rear wheels of the electric bus according to the desired yaw moment constraint.

### Desired Yaw Moment Calculation Based on Neural Network PID Controller

The traditional PID control technology has a large steady-state error for the complex vehicle control system due to the single control parameter. Compared with tradition PID control the neural network PID controller corrects the neural network weights in real time by obtaining the errors of the side-slip angle and the yaw rate of the actual vehicle and the reference model. Therefore, the neural network PID controller does not need to generate training data in advance to train the neural network, which greatly shortens the initialization time of the neural network.

**FIGURE 4:**
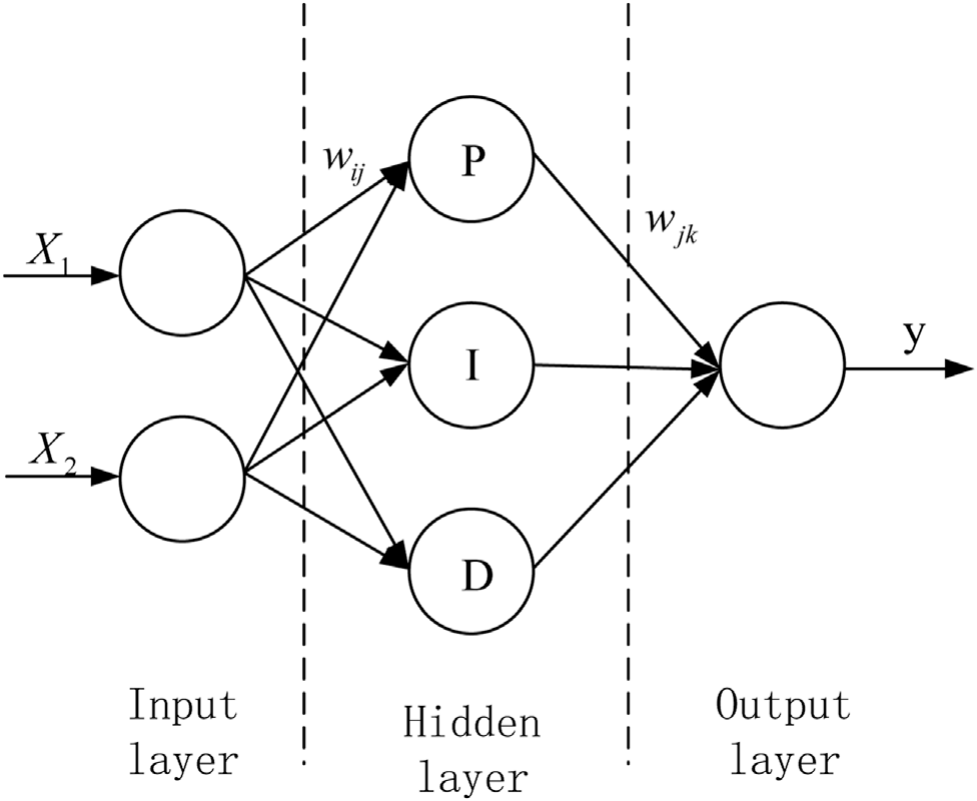
Topological structure of neural network PID sub-network.

The adopted neural network PID controller has a three-layer forward network structure, including input layer, hidden layer and output layer, and its sub-network topology is shown in Figure 4.

The inputs X1 and X2 of the input layer are the actual value and target value of the control variable, respectively, and the output Y of the output layer is the calculated control law. The neural network calculates the error according to the objective function, uses the gradient correction method to continuously correct the network weights of each layer, calculates the neuron input of each layer, and then performs iterative update operation according to each neuron update method, and finally obtains a suitable controller.

**FIGURE 5:**
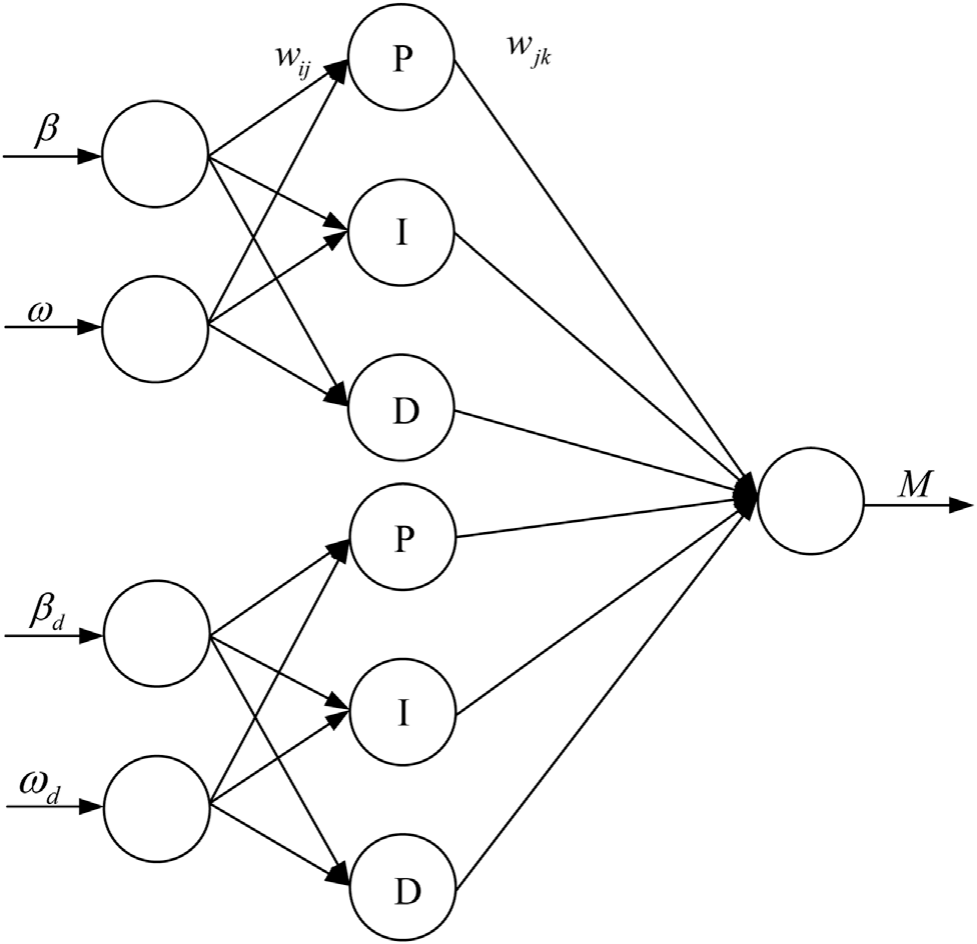
Topology diagram of direct yaw moment control strategy.

The topology of the neural network PID algorithm designed for the stability of the vehicle in this paper is shown in Figure 5.

The objective function of the neural network weight correction in this paper is

$$J=\min\left[{\left(\omega -\omega_{d}\right)}^{2}+{\left(\beta -\beta_{d}\right)}^{2}\right]$$ (3.1)

Because the gradient learning algorithm is easy to fall into the local optimum, the method of increasing the momentum term can improve the network learning efficiency. The weight update formula is:

$$w_{j}^{\left(z\right)}=w_{j}^{\left(z-1\right)}-\eta \frac{\partial J}{\partial w_{j}^{\left(z-1\right)}}+\eta_{1}\left(w_{j}^{\left(z-1\right)}-w_{j}^{\left(z-2\right)}\right)$$ (3.2)

$$w_{ij}^{\left(z\right)}=w_{ij}^{\left(z-1\right)}-\eta \frac{\partial J}{\partial w_{ij}^{\left(z-1\right)}}+\eta_{1}\left(w_{ij}^{\left(z-1\right)}-w_{ij}^{\left(z-2\right)}\right)$$ (3.3)

In the formula, *w*_*j*_ is the weight from the hidden layer to the output layer; *w*_*ij*_ is the weight from the input layer to the hidden layer; the superscript z represents the z ^th^ optimization, the *z-1*st optimization weight and the *z-2*nd optimization weight. The difference between the values ​​is the momentum term, z is greater than 2; *η*and *η*_*1*_are the learning rates.

The input of the input layer is the reference value and the actual value of the center of mass slip angle and yaw angular velocity, X is the input matrix of the input layer, which is:

$$X={\left[\begin{array}{cccc} x_{1} & x_{2} & x_{3} & x_{4} \end{array}\right]}^{T}={\left[\begin{array}{cccc} \omega & \omega_{d} & \beta & \beta_{d} \end{array}\right]}^{T}$$ (3.4)

The hidden layer has 6 neurons, the input matrix of the hidden layer is net, j is the number of neurons in the hidden layer, and wij is the weight from the input layer to the hidden layer. The formula for the input value of the hidden layer is:

$$\left\{ \begin{array}{l} net_{j}=\sum_{i=1}^{2}x_{i}w_{ij},j=1,2,3\\ net_{j}=\sum_{i=3}^{4}x_{i}w_{ij},j=4,5,6 \end{array}\right.$$ (3.5)

Let the output of the hidden layer be netout. According to the different functions of each neuron in the hidden layer, the outputs of various types of neurons are designed as:

$$net_{out}\left(j\right)=net_{j},\;j=1,4$$ (3.6)

$$net_{out}\left(j\right)=net_{j}+net_{j}^{*},\;j=2,5$$ (3.7)

$$net_{out}\left(j\right)=net_{j}-net_{j}^{*},\;j=3,6$$ (3.8)

In the formula, netj* is the neuron output value calculated by the previous operation step in the calculation process.

The output layer has a neuron whose output value is the control rate Mz, and its calculation formula is:

$$M_{z}=\sum_{j=1}^{6}net_{out}\left(j\right)w_{j}$$ (3.9)

where *w*_*j*_ is the weight from the hidden layer to the output layer.

### Driving Torque Distribution

After the total driving torque $T_{d}$ is determined by the accelerator pedal, the driving torque needs to be reasonably distributed to the left and right rear driving wheels. When the vehicle is straight driving, the total driving torque $T_{d}$ is equally distributed to the left and right driving wheels. When the vehicle turns, the torques of the left and right drive wheels are no longer equal, and satisfy the constraint of desired yaw moment ***M*** calculated by the neural network PID control.

By analyzing the driving state of the vehicle, it can be seen that when the vehicle understeer on the left or oversteer on the right, in order to ensure the steering stability of the vehicle, the torque of the right wheel should be increased and the torque of the left wheel should be reduced; When the left side of the vehicle is oversteering or the right side is understeering, the torque of the left wheel shall be increased and the torque of the right wheel shall be reduced to ensure the steering stability of the vehicle. Therefore, the torques of left and right driving wheels should meet the constraints of the following Eq. 3.10

$$\{\begin{array}{c} T_{RR}+T_{RL}=T_{d}\\ \left(T_{RR}-T_{RL}\right)\frac{W}{2R}=M \end{array}$$ (3.10)

In the above formula, W is the rear wheel thread, and R is the rolling radius of the driving wheel.

## Simulation Analysis

**FIGURE 6:**
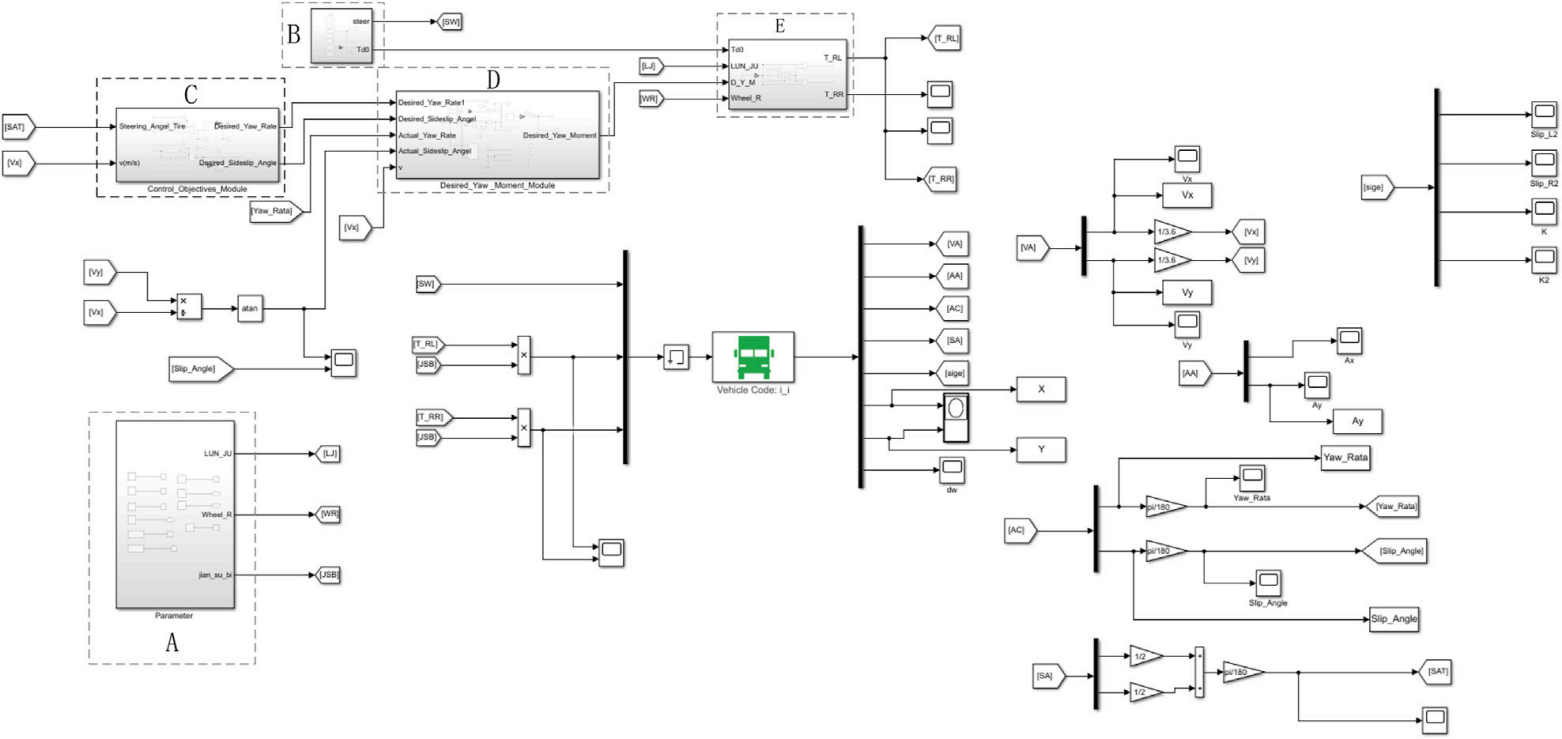
Matlab/Simulink-TruckSim co-simulation platform.

Based on the distributed rear-wheel drive electric bus model, two typical driving conditions of double line shifting condition and slalom test at medium speed are selected to verify and analyze the control effect of the designed distributed drive control strategy. At the same time, it is compared with vehicles without control and ordinary PID control. According to the above analysis and design, the Matlab/Simulink software and TruckSim software are used to establish a co-simulation platform, shown in Figure 6. The name, input and output of each module are shown in Table 2.

**TABLE 2:** Name, input and output of each module.

The Module Number	The name of Module	Input	Output
A	Vehicle parameters	No	Wheel thread, tire radius, deceleration ratio
B	Driver model	No	Total drive torque, steering angle
C	Two-degrees-of-freedom reference model	Front wheel average turning angle, speed	Desired yaw rate, desired sideslip angle
D	The neural network PID controller model	Desired yaw rate, desired sideslip angle, actual yaw rate, actual sideslip angle	Additional yaw-moment
E	The driving torque allocation model	Total drive torque, wheel thread, tire radius, additional yaw-moment	Left rear wheel driving torque, right rear wheel driving torque

### Double Line Shifting Condition

**FIGURE 7:**
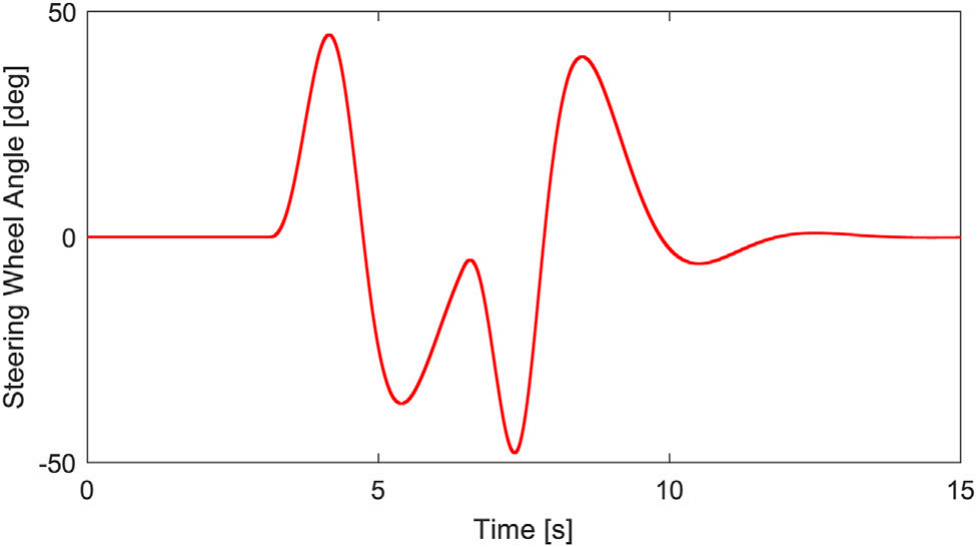
Steering wheel angle.

**FIGURE 8:**
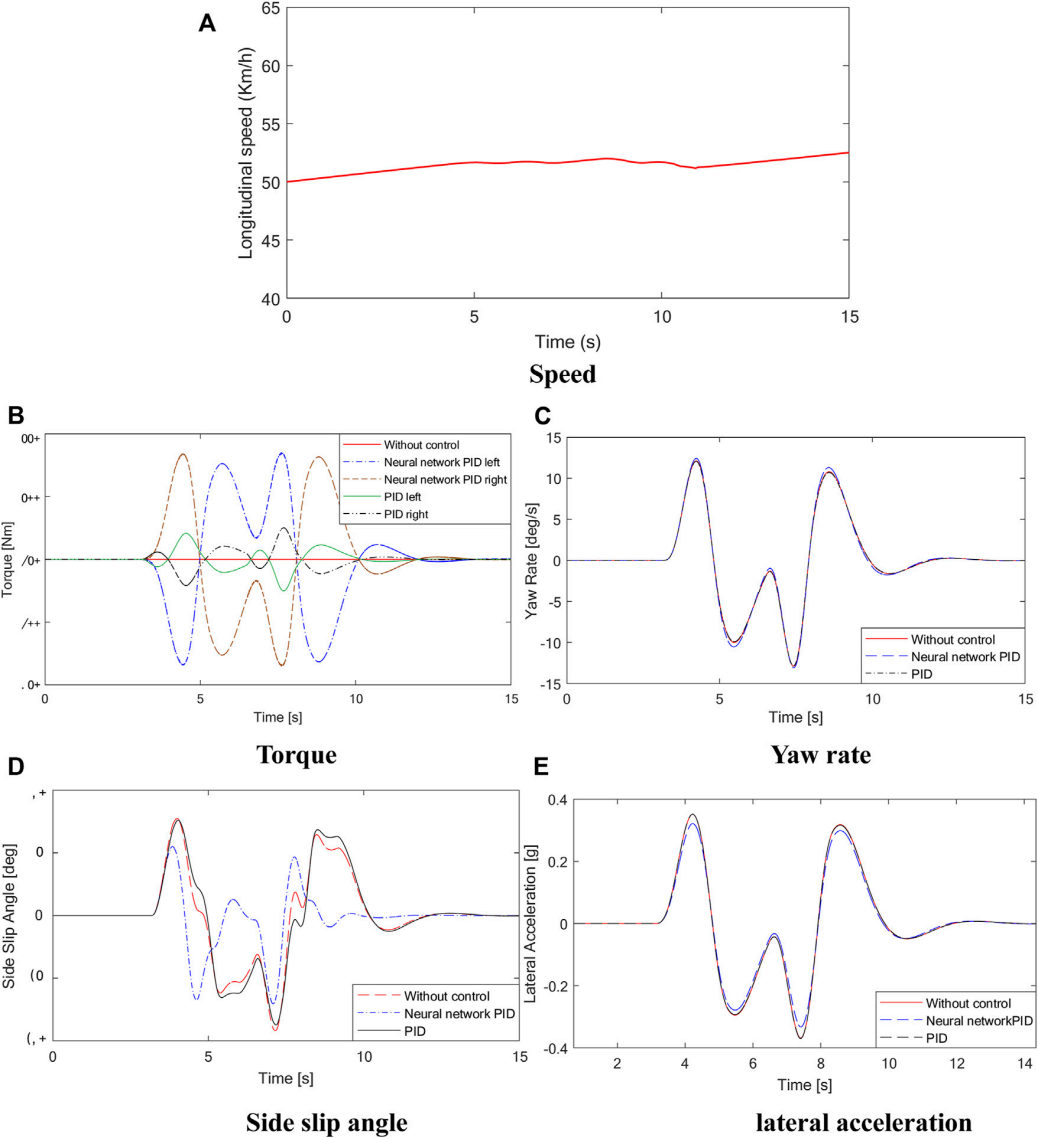
Simulation results. **(A)** is the longitudinal speed curve, **(B)** is the torque curve, **(C)** is the yaw rate curve, **(D)** is the side slip angle curve, **(E)** is the lateral acceleration curve.

The driving of a car is inseparable from the operation of the driver, and the double-shift test is a typical working condition of the human-vehicle closed-loop system. It is generally believed that the double-shift test condition is used to simulate emergency risk aversion and lane overtaking. In this paper, the medium-speed double-moving line condition is selected to simulate the emergency situation. Assuming that the road adhesion coefficient of the vehicle on the road is μ = 0.7, and the driving speed is 50 km/h, the driver operates the steering wheel to avoid danger when he encounters a driving obstacle after 5 s. In this process, the speed is generally kept constant. Steering wheel angle signal, speed variation curve, wheel torque output curve, yaw angular velocity curve, centroid yaw angle curve and lateral acceleration curve are shown in Figure 7, Figure 8.

As shown in Figure 8, there is a torque difference between the left and right driving wheels of the vehicle using conventional PID control and neural network PID control strategy during the test of the vehicle under double-shift conditions. However, under the conventional PID control strategy, the torque difference between the left and right driving wheels is small, and the yaw angular velocity, center of mass yaw angle and lateral acceleration curves in Figure 8 also show that the values of the conventional PID control strategy are almost the same as those of the non-control group. The maximum value of the centroid side slip angle is 9deg without control, while under the neural network PID control strategy, the maximum mass center side slip angle is 6deg, and the mass center side slip angle can be effectively reduced by 30%, indicating that the control effect of the electronic differential control program is obvious, at the same time, the peak lateral acceleration of the vehicle can also be effectively suppressed, and the lateral stability margin is improved. The simulation results show that the designed neural network PID control strategy can effectively improve the stability of vehicles under double-shift conditions.

### Slalom Test at Medium Speed Driving Condition

**FIGURE 9:**
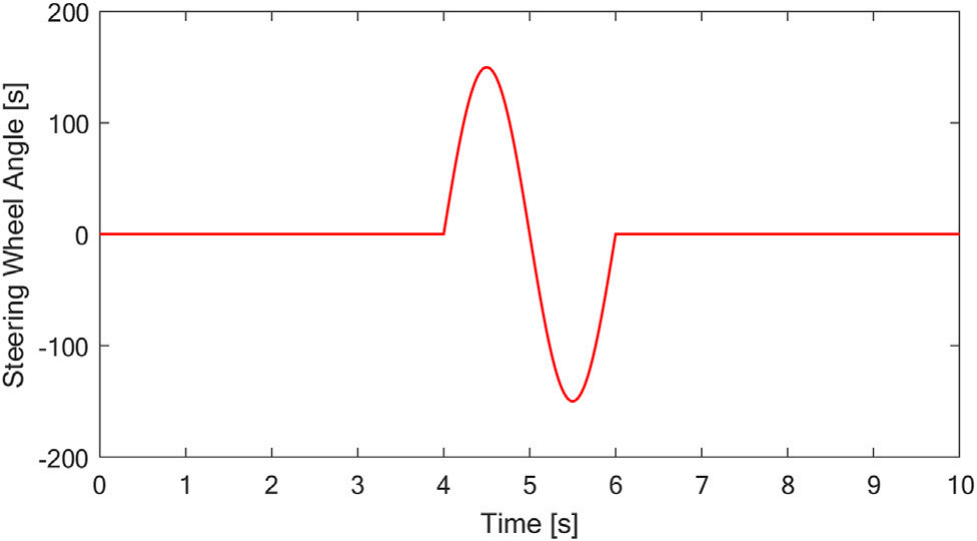
Steering wheel angle.

**FIGURE 10:**
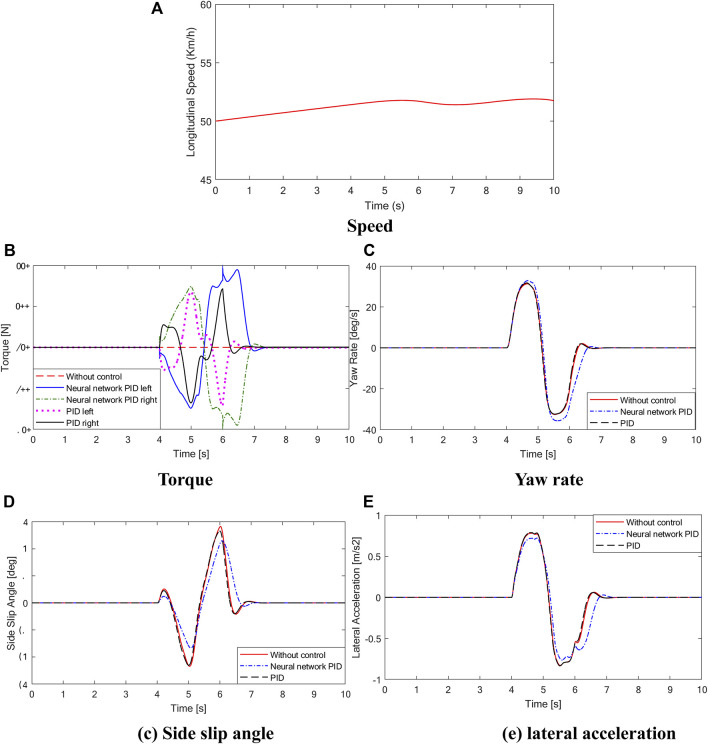
Simulation results. **(A)** is the longitudinal speed curve, **(B)** is the torque curve, **(C)** is the yaw rate curve, **(D)** is the side slip angle curve, **(E)** is the lateral acceleration curve.

The driving condition of the vehicle with a slalom test at medium speed on a low adhesion coefficient road is simulated. Assuming that the vehicle is driving on a road with an adhesion coefficient of *μ* = 0.7, the initial speed is 50 km/h, the steering wheel performs 150° reciprocating steering operation after 4s, and the steering signal is shown in Figure 9. The curves of vehicle speed, yaw rate, and sideslip angle are shown in Figure 10.

As shown in Figure 10, during the sinusoidal shift test of the vehicle, the steering wheel angle of the vehicle changes from 0 to 150 deg in the process of 4–6 s and then to −150 deg and finally back to 0 deg. There is a large torque difference between the left and right driving wheels of the vehicle with conventional PID control and neural network PID control strategy. The results show that the differential effect of the controller is obvious under the condition of rapid change of steering wheel. The torque diagram in Figure 10A shows that under conventional PID control, the output torque of the vehicle changes sharply, which is easy to cause body jitter. At the same time, the comparison curves of yaw velocity, centroid yaw angle and lateral acceleration show that under the neural network PID control strategy, the state parameters of the vehicle are the best, and the maximum value of the centroid yaw angle is 8.7deg without control, while under the neural network PID control strategy, the maximum value of the centroid yaw angle is 6.2deg, and the centroid yaw angle can be effectively reduced by 28%. The simulation results show that the centroid side slip angle can be effectively reduced by 28%. The designed neural network PID control strategy can effectively improve the stability of the vehicle under the sinusoidal moving condition.

## Conclusion

In this paper, the distributed drive control strategy was proposed. Based on the direct yaw moment control principle and the advantages of independent controllable driving torque of the distributed rear-drive electric bus, the neural network PID controller was designed to calculate the additional yaw moment, and the optimal driving torques of the left and right rear wheels were obtained through the driving torque distribution rules. two typical driving conditions of double line shifting condition and slalom test at medium speed were carried out and analyzed by Matlab/Simulink-TruckSim co-simulation. The simulation results show that the designed neural network PID control strategy can effectively reduce the sideslip angle and lateral acceleration of the vehicle center of mass and improve the stability of the vehicle.

In the future, hardware-in-the-loop (HIL) tests will be performed to further verify the designed control strategy.

## Data Availability

The original contributions presented in the study are included in the article/Supplementary Material, further inquiries can be directed to the corresponding author.
